# “Seeing Is Believing”: Additive Utility of ^68^Ga-PSMA-11 PET/CT in Prostate Cancer Diagnosis

**DOI:** 10.3390/cancers16091777

**Published:** 2024-05-05

**Authors:** Joel Chin, Yu Guang Tan, Alvin Lee, Tze Kiat Ng, Ruoyu Shi, Charlene Yu Lin Tang, Sue Ping Thang, Jeffrey Kit Loong Tuan, Christopher Wai Sam Cheng, Kae Jack Tay, Henry Sun Sien Ho, Hung-Jen Wang, Peter Ka-Fung Chiu, Jeremy Yuen-Chun Teoh, Winnie Wing-Chuen Lam, Yan Mee Law, John Shyi Peng Yuen, Kenneth Chen

**Affiliations:** 1 Department of Urology, Singapore General Hospital, Outram Road, Singapore 169608, Singapore; 2 Department of Anatomical Pathology, Singapore General Hospital, Outram Road, Singapore 169608, Singapore; 3 Department of Nuclear Medicine and Molecular Imaging, Singapore General Hospital, Outram Road, Singapore 169608, Singapore; 4 Division of Radiation Oncology, National Cancer Centre Singapore, 11 Hospital Drive, Singapore 169610, Singapore; 5 SingHealth Duke-NUS Oncology Academic Clinical Programme, 20 College Rd, Singapore 169856, Singapore; 6 Department of Urology, Kaohsiung Chang Gung Memorial Hospital, Chang Gung University College of Medicine, Kaohsiung 807, Taiwan; 7 S.H. Ho Urology Centre, Department of Surgery, The Chinese University of Hong Kong, Hong Kong, China; 8 SingHealth Duke-NUS Radiological Sciences Academic Clinical Programme, 20 College Rd, Singapore 169856, Singapore; 9 Department of Diagnostic Radiology, Singapore General Hospital, Outram Road, Singapore 169608, Singapore; 10 SingHealth Duke-NUS Surgery Academic Clinical Programme, 20 College Rd, Singapore 169856, Singapore

**Keywords:** prostate cancer diagnosis, mpMRI, PSMA PET, biopsy

## Abstract

**Simple Summary:**

This study investigates the effectiveness of combining two imaging techniques, multiparametric magnetic resonance imaging (mpMRI) and ^68^Ga-Prostate-specific membrane antigen (PSMA-11) positron emission tomography/computed tomography (PET/CT), to diagnose clinically significant prostate cancer (csPCa). While mpMRI is commonly used, it has limitations in its accuracy, requires further confirmation with prostatic biopsy. This study explores whether adding ^68^Ga-PSMA-11 PET/CT enhances diagnostic accuracy. The results show that the combined approach significantly improves the detection of csPCa compared to using either modality alone. Specifically, when both imaging methods are able to detect suspicious lesions, the likelihood of csPCa is high. This study suggests that, in select cases with convincing imaging results, it may be possible to forgo biopsy before surgical treatment. However, further research is needed to validate these findings and develop predictive models for accurate diagnosis without biopsy.

**Abstract:**

Widespread adoption of mpMRI has led to a decrease in the number of patients requiring prostate biopsies. ^68^Ga-PSMA-11 PET/CT has demonstrated added benefits in identifying csPCa. Integrating the use of these imaging techniques may hold promise for predicting the presence of csPCa without invasive biopsy. A retrospective analysis of 42 consecutive patients who underwent mpMRI, ^68^Ga-PSMA-11 PET/CT, prostatic biopsy, and radical prostatectomy (RP) was carried out. A lesion-based model (*n* = 122) using prostatectomy histopathology as reference standard was used to analyze the accuracy of ^68^Ga-PSMA-11 PET/CT, mpMRI alone, and both in combination to identify ISUP-grade group ≥ 2 lesions. ^68^Ga-PSMA-11 PET/CT demonstrated greater specificity and positive predictive value (PPV), with values of 73.3% (vs. 40.0%) and 90.1% (vs. 82.2%), while the mpMRI Prostate Imaging Reporting and Data System (PI-RADS) 4–5 had better sensitivity and negative predictive value (NPV): 90.2% (vs. 78.5%) and 57.1% (vs. 52.4%), respectively. When used in combination, the sensitivity, specificity, PPV, and NPV were 74.2%, 83.3%, 93.2%, and 51.0%, respectively. Subgroup analysis of PI-RADS 3, 4, and 5 lesions was carried out. For PI-RADS 3 lesions, ^68^Ga-PSMA-11 PET/CT demonstrated a NPV of 77.8%. For PI-RADS 4–5 lesions, ^68^Ga-PSMA-11 PET/CT achieved PPV values of 82.1% and 100%, respectively, with an NPV of 100% in PI-RADS 5 lesions. A combination of ^68^Ga-PSMA-11 PET/CT and mpMRI improved the radiological diagnosis of csPCa. This suggests that avoidance of prostate biopsy prior to RP may represent a valid option in a selected subgroup of high-risk patients with a high suspicion of csPCa on mpMRI and ^68^Ga-PSMA-11 PET/CT.

## 1. Introduction

There is growing interest in utilizing image guidance to detect clinically significant prostate cancer (csPCa), defined as lesions scoring as International Society of Urological Pathology (ISUP) grade group ≥ 2. Multiparametric MRI (mpMRI) has emerged as a crucial tool in PCa diagnostics, drawing considerable attention for its commendable diagnostic accuracy in the screening for significant PCa [1]. While newer imaging sequences and magnets with greater strength have been able to produce images with better quality, mpMRI still faces limitations, notably its variable positive predictive values (PPV) of 34–68%, and mixed outcomes of its use have been observed [1]. The landmark PROMIS trial found that a negative mpMRI could spare prostate biopsies in 27% of patients, but its specificity and PPV for csPCa were constrained, resulting in missed diagnoses of PCa [2]. The subsequent PRECISION trial corroborated these findings [3]. Even with a Prostate Imaging Reporting and Data System (PI-RADS) maximum score of 5, 17% of the biopsies performed had the results come back as insignificant PCa or benign tissue, thus underscoring the inadequacy of mpMRI alone to definitively confirm PCa without histological biopsy [3].

^68^Ga-Prostate-specific membrane antigen (PSMA-11) positron emission tomography/computed tomography (PET/CT) is a valuable diagnostic tool for imaging prostate cancer that exhibits elevated levels of prostate-specific membrane antigen (PSMA), also known as glutamate carboxypeptidase II [4]. PSMA is a transmembrane protein predominantly expressed in all prostate tissues [4]. Its expression is significantly increased in prostate cancer cells, with significantly higher levels of expression in de-differentiated, metastatic, or castration-resistant disease [5]. Comparative studies have demonstrated that ^68^Ga-PSMA-11 PET/CT surpasses conventional imaging methods like CT, MRI, and bone scans, postulating that ^68^Ga-PSMA-11 PET/CT should be prioritized as a first-line approach for the initial staging of intermediate- to high-risk PCa [6]. Due to its increased sensitivity and specificity, ^68^Ga-PSMA-11 PET/CT has gained approval from the United States Food and Drug Administration for both the initial diagnosis and staging of suspected metastases in patients with prostate cancer, as well as for the imaging of individuals suspected of experiencing biochemical recurrence post-prostatectomy or radiation therapy [7]. 

The recent PRIMARY trial expounded on the added value of ^68^Ga-PSMA-11 PET/CT in identifying lesions indicative of csPCa during the initial diagnostic phase, demonstrating improved negative predictive value and sensitivity when used in conjunction with mpMRI [8]. Prostatic lesions exhibiting a high maximum standardized uptake value (SUVmax) of 12 or higher on ^68^Ga-PSMA-11 PET/CT and a PI-RADS score greater than 4 on mpMRI are highly indicative of csPCa [9]. Consequently, the authors proposed that such patients might potentially be able to skip confirmatory biopsy and proceed directly to definitive therapy. This raises the consideration as to whether histological confirmation of suspected lesions remains necessary when both imaging modalities are employed together [10]. Such a diagnostic approach could potentially reduce the considerable number of prostate biopsies conducted annually in specific patient cohorts [11]. Similar practices of therapy initiation without histological confirmation already exist for other malignancies, such as renal cell carcinoma and hepatocellular carcinoma, where histological proof is reserved for radiologically indeterminate masses [12]. By omitting prostatic biopsy from the diagnostic pathway for a selected cohort of patients, this may potentially result in a lower burden for the patient, as even the transperineal approach carries the risk of complications [13]. 

This study aims to evaluate the diagnostic accuracy of ^68^Ga-PSMA-11 PET/CT and mpMRI in detecting csPCa and determine whether ^68^Ga-PSMA-11 PET/CT has additive value for the diagnostic ability of mpMRI.

## 2. Methods

We reviewed our institution’s prospectively maintained cancer registry and selected men who had undergone both radical prostatectomy (RP) from 2020 to 2022 for biopsy-proven cancer and pre-biopsy mpMRI and ^68^Ga-PSMA-11 PET/CT for initial diagnosis and staging. A total of 42 consecutive men were identified and found to be suitable for analysis.

### 2.1. PET/CT Imaging

The administered radioactivity of ^68^Ga-PSMA-11 ranged from 104 MBq to 152 MBq. Following the department’s protocol [14], patients underwent scanning with a delay of 60 to 75 min after the tracer injection. CT scans were performed for attenuation correction and anatomical correlation. The scanning procedure covered from the top of the skull to the upper thighs. PET data were acquired using 3-dimensional time-of-flight (TOF) mode, with a duration of 2 min per bed position and 5–6 bed positions per patient, with a 25% overlap, varying based on the patient’s size. 

The datasets were independently reviewed by two board-certified nuclear medicine physicians—one with at least 2 years’ experience and another with more than 10 years’ experience. The readers were blinded to all clinical information. In cases of discrepancy, consensus was obtained by joint reading. The region of interest (ROI) was manually delineated as a region exhibiting an anomalous signal in MRI sequences or displaying positive lesions on ^68^Ga-PSMA-11 PET/CT. There was no maximum standardized uptake value cutoff used to define a positive result. A localized abnormality within the prostate displaying uptake levels surpassing the background uptake, not ascribable to physiologic processes, was deemed positive on ^68^Ga-PSMA-11 PET. The SUVmax was quantified based on ROI.

### 2.2. MRI

All patients underwent 3-Tesla mpMRI (Magnetom Skyra, Siemens Healthineers, Pittsburgh, PA, USA) performed using our institution’s PI-RADs v2.1-compliant standard protocol [15]. In brief, the protocol included obtaining high-resolution, multiplanar, small-field-of-view (FOV), T2-weighted turbo-spin-echo images in axial, sagittal, and coronal planes; high B-value diffusion weighted imaging (DWI B-values 0, 500, 1000, 1800 s/mm^2^); and dynamic contrast-enhanced imaging (DCE temporal resolution 4.47 s) following a dose of gadolinium contrast agent (Gadovist, Schering AG, Berlin, Germany). These imaging sequences were acquired with a 60-channel pelvic phase array coil. Additionally, apparent diffusion coefficient mapping was performed with all B-values. A genitourinary radiologist with 9 years’ experience reading prostate mpMRIs who was blinded to the biopsy and surgical outcomes retrospectively reviewed all mpMRIs using PI-RADs v2.1 criteria [16]. Prostate boundaries and suspicious lesions were marked using Urofusion (BioBot Surgical Ltd., Singapore) software to produce a 3D MRI model. All lesions detected were assigned a lesion-specific PI-RADS category score. All lesions assigned PI-RADS ≥ 3 were deemed positive.

### 2.3. Histopathology

Each radical prostatectomy specimen was fixed in formalin and subjected to a standardized fixation regime for 48 h prior to further processing. Within each specimen, every cancer focus was carefully marked out with indelible ink (Staedtler Lumocolor fine-tip marker 0.6 mm width) on 3.5 mm slices. Malignant focuses located less than 1 mm apart within the same plane were regarded as part of the same lesion. All whole-mount histology sections were reviewed by a senior uropathologist with 8 years of experience in genitourinary pathology and reported according to the College of American Pathologists protocol [17]. CsPCa was defined as ISUP grade group 2 and above, identified on whole-mount RP specimen. Lesions were localized in quadratic fashion in the axial dimension and trisectioned in the coronal dimension (base, mid, and apex) according to respective modality. If there was a discrepancy within each segmented location (e.g., 2 lesions at right posterior base on the whole mount instead of 1 lesion seen on MRI at the same segment), digital overlays of the axial images were used to arbitrate. 

### 2.4. Statistical Analyses

Patient characteristics were compared using descriptive analysis. Logistic regression analysis, incorporating receiver-operating characteristic curves and area under the curve (AUC) analysis, was performed to investigate the relationship between mpMRI, ^68^Ga-PSMA-11 PET/CT, and both modalities in tandem (mpMRI and ^68^Ga-PSMA-11 PET/CT) in terms of detecting csPCa using a lesion-based approach. A total of 122 lesions (drawn from mpMRI, ^68^Ga-PSMA-11 PET/CT, and whole-mount histopathology) were analyzed from 42 eligible patients. The sensitivity, specificity, PPV, and NPV of the various imaging modalities were calculated. A *p* value of <0.05 was taken to indicate statistical significance. Analysis was performed using SPSS Statistics for Windows, Version 25.0 (IBM, Armonk, NY, USA). 

## 3. Results

The patient and lesion characteristics are summarized in Table 1. The median age of patients was 69 years old (IQR 65.8–71.3). The median PSA at diagnosis was 12.0 ng/mL (IQR 6.08–24.3), and the median prostate volume was 46 mL (IQR 31.9–58.0), thus arriving at a calculated median PSA density of 0.253 ng/ml/ml (IQR 0.17–0.67). Histopathological evaluation of the RP specimens showed csPCa in all patients. Index lesion Gleason grade groups were mostly Gleason 3 + 4 = 7 (*n* = 15, 35.7%), Gleason 4 + 3 = 7 (*n* = 17, 40.5%), and ≥Gleason 4 + 4 = 8 (*n* = 10, 23.8%).

A lesion-based analysis showed a total of 122 lesions identified on mpMRI, ^68^Ga-PSMA-11 PET/CT, and whole-mount histopathology. Final whole-gland histopathology demonstrated 92 (75.4%) lesions associated with ISUP grade group ≥ 2 PCa, 8 correlating with ISUP grade group 1 PCa, and 22 with no cancer correlate. mpMRI picked up all but 3 lesions, with the following distribution in terms of PI-RADS scores: 18 PI-RADS 3, 65 PI-RADS 4, and 36 PI-RADS 5. Of the 122 lesions analyzed, 80 lesions were positive on the ^68^Ga-PSMA-11 PET/CT, while 42 lesions were not. 

The diagnostic accuracy of the various imaging modalities is shown in Table 2, with the AUC values and measures of sensitivity, specificity, NPV, and PPV. Assessing ISUP grade group ≥ 2 PCa using lesion-based analysis, mpMRI (PI-RADS 4–5) demonstrated a sensitivity of 90.2%, specificity of 40.0%, PPV of 82.2%, NPV of 57.1%, and AUC of 0.65. The sensitivity, specificity, PPV, NPV, and AUC of ^68^Ga-PSMA-11 PET/CT were 78.5%, 73.3%, 90.1%, 52.4%, and 0.76, respectively. The combination of mpMRI (PI-RADS 4–5) and ^68^Ga-PSMA-11 PET/CT further improved the diagnosis of csPCa, with sensitivity, specificity, PPV, NPV, and AUC of 74.2%, 83.3%, 93.2%, 51%, and 0.79, respectively.

Subgroup analysis of PI-RADS 3, 4, and 5 lesions were carried out to evaluate the additive value of ^68^Ga-PSMA-11 PET/CT. In PI-RADS 3 lesions, ^68^Ga-PSMA-11 PET/CT achieved sensitivity, specificity, PPV, and NPV of 66.7%, 70.0%, 57.1%, and 77.8%. In PI-RADS 4 lesions, these values were 76.2%, 56.3%, 82.1%, and 47.4%. In PI-RADS 5 lesions, ^68^Ga-PSMA-11 PET/CT scored 100% throughout. Of the 37 PI-RADS 5 lesions, 36 contained csPCa and were positive on ^68^Ga-PSMA-11 PET/CT. The one PI-RADS 5 lesion which showed benign whole-mount histology was negative on a ^68^Ga-PSMA-11 PET/CT scan.

**Figure 1:**
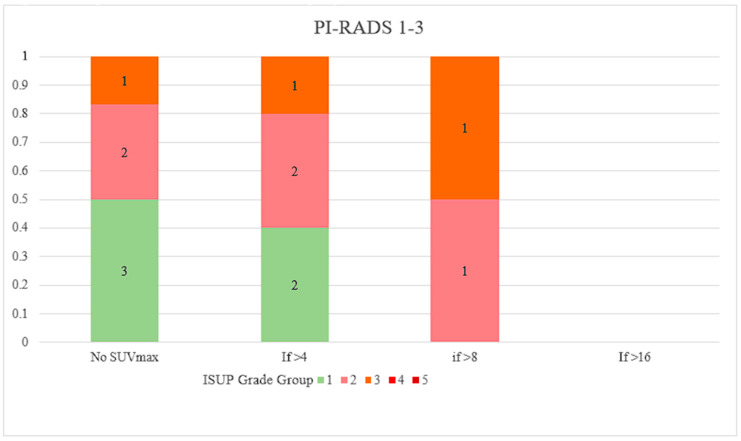
Proportion of PCa ISUP Grade Groups by SUVmax thresholds for PI-RADS 1–3 Lesions. PCa, prostate cancer; ISUP, International Society of Urological Pathology; SUVmax, maximum standardised uptake value; PI-RADS, Prostate Imaging-Reporting and Data System.

**Figure 2:**
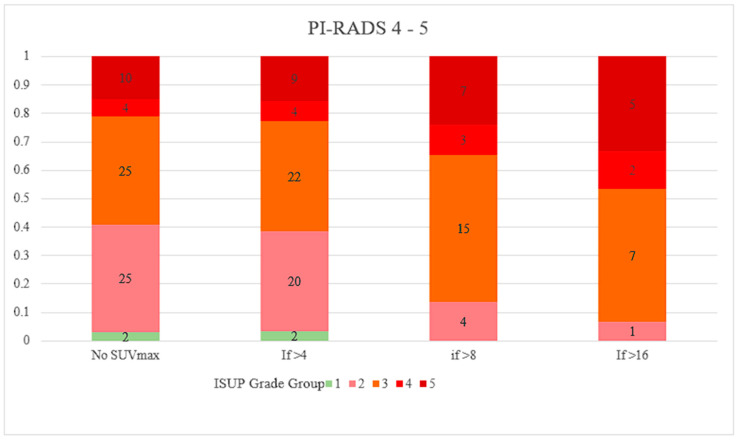
Proportion of PCa ISUP Grade Groups by SUVmax thresholds for PI-RADS 4–5 Lesions. PCa, prostate cancer; ISUP, International Society of Urological Pathology; SUVmax, maximum standardised uptake value; PI-RADS, Prostate Imaging-Reporting and Data System.

Figure 1 and Figure 2 shows the relative proportions of PCa in different ISUP grade groups plotted against SUVmax thresholds for the PI-RADS 1–3 and 4–5 groups. Out of the 31 patients with SUVmax > 8, regardless of PI-RADS score, all had ISUP grade group ≥ 2 disease, and 83.9% of them had ISUP grade group ≥ 3 disease. The combination of PI-RADS 4 or 5 with SUVmax > 4 resulted in ISUP grade group ≥ 2 disease at a rate of 96.5%.

## 4. Discussion

There is increasing interest in leveraging the use of novel imaging modalities to detect csPCa. The concept of replacing invasive procedures like conventional prostate biopsies using non-invasive imaging methods to accurately identify csPCa is appealing. However, achieving a high level of sensitivity and specificity is imperative before such modalities can be widely adopted in clinical practice. 

Among the various imaging modalities, mpMRI has emerged as a pivotal tool in the detection, localization, local staging, and management of csPCa [18]. Nevertheless, its efficacy is hampered by its variable PPV, ranging from 34% to 68% [2]. To tackle this issue, molecular imaging approaches, such as employing ^68^Ga-PSMA-11 PET/CT, have been suggested. Currently, ^68^Ga-PSMA-11 PET/CT is primarily being used for the distant staging of high-risk PCa following biopsy or for staging biochemical recurrence after local treatment [19].

The current diagnostic pathway recommends prostate biopsy for men considered at risk due to an elevated PSA and/or abnormal digital rectal examination findings. If a pre-biopsy mpMRI detects suspicious or equivocal lesions, further targeted biopsy is conducted [20]. However, prostate biopsy, especially with conventional transrectal ultrasound biopsy, carries potential morbidity, prompting many patients to seek less invasive options [21]. Recently, a large-scale national study from the United Kingdom revealed that infectious complications such as sepsis (<1.5%), urinary retention (<2%), and hematuria requiring catheterization (<1%) were potential morbidities associated with both transperineal and transrectal biopsy [21]. This raises the question whether it is feasible, in selected cases, to avoid unnecessary biopsies before proceeding to local treatment with RP in cases of highly suspicious imaging results.

In this study, we sought to explore the feasibility of predicting csPCa with a combination of pre-biopsy mpMRI and ^68^Ga-PSMA-11 PET/CT results in a surgical cohort of patients undergoing RP.

Every participant in the study had intermediate-risk PCa confirmed through biopsy and managed with RP. Consequently, it is not surprising that almost all men showed positive findings on both mpMRI and ^68^Ga-PSMA-11 PET/CT when assessed through a whole-gland analysis. However, the main aim of this study was to ascertain ^68^Ga-PSMA-11 PET/CT’s ability to discriminate individual lesions harboring significant PCa and those containing no or insignificant PCa histology. Hence, the value of our findings is rooted in the detailed per-lesion analyses. As seen from this study, there is an increase in PPV and complementary effectiveness of ^68^Ga-PSMA-11 PET/CT compared with and in addition to mpMRI for detecting csPCa. Utilizing both mpMRI (PI-RADS 4–5) and ^68^Ga-PSMA-11 PET/CT demonstrated high specificity and PPV (83.3% and 93.2%, respectively) for the detection of ISUP grade group ≥ 2 PCa. When further coupled with an SUVmax cut-off of 4, this increased the detection rate of csPCa to 96.5%. These results illustrate that the PSMA activity on ^68^Ga-PSMA-11 PET/CT is highly indicative of the presence of csPCa.

This concept of using ^68^Ga-PSMA-11 PET/CT to supplement mpMRI for primary diagnosis is becoming more relevant, given that ^68^Ga-PSMA-11 PET/CT has undisputed superior accuracy over conventional imaging in the distant staging of intermediate- to high-risk prostate cancer [6]. Hence, as the indication for an upfront ^68^Ga-PSMA-11 PET/CT strengthens and continues to expand, its added utility in the confirmation of primary tumors may have potential to omit biopsies in selected cases with compelling findings on dual imaging. This has multiple benefits; not only does this prevent the potential morbidities associated with invasive biopsy, but it also translates to cost savings for patients and healthcare providers, and also potentially resolves issues related to resource limitations. In very high-risk cases, this would also reduce the delay in initiating definitive treatment caused by the intermediary step of a prostate biopsy.

The obtained results are biologically consistent, as they align with our existing understanding of the PSMA receptor. PSMA is a transmembrane glycoprotein predominantly expressed on the cell surface of PCa cells at significantly elevated levels compared to normal cells [4]. The density of PSMA receptors tends to be positively correlated with the aggressiveness of PCa cells and is particularly elevated in castrate-resistant cells [5]. 

The primary limitation of our study stems from its retrospective design and the consequent selection bias inherent in a surgical cohort. Patients were retrospectively identified from a prospectively maintained database, all of whom underwent RP. This method of recruitment introduced a selection bias, as all patients enrolled in the study had biopsy-confirmed significant PCa. Whilst our findings demonstrate a high sensitivity for csPCa in this high-prevalence setting, further assessment in a prospective cohort with a lower prevalence of csPCa is warranted to further ascertain if it has any clinical role in reducing biopsy in men with suspected cancer. 

While the use of a cohort of patients selected for radical prostatectomy may skew the diagnostic characteristics, the inclusion of whole-mount histology data offers the best veracity regarding the presence, location, and Gleason grade of cancer foci. The utilization of whole-mount histology allows for a meticulous correlation with pre-operative imaging findings, offering insights into the concordance between scan results and the true extent of disease. Evidence has shown a notable discordance between cancer foci identified through biopsy and those seen in whole-mount histology, with close to a quarter of patients exhibiting additional lesions with a Gleason score of 7 or higher on final whole-mount sections [22]. Thus, we assert that the enhanced accuracy afforded by the usage of whole-mount histology was crucial for our lesion-based analysis. By leveraging this comprehensive dataset, we aimed to provide a more nuanced understanding of the diagnostic performance of pre-operative imaging modalities in identifying csPCa.

Conducting initial ^68^Ga-PSMA-11 PET/CT scans for all patients to decide on the indication for biopsy may not currently be deemed cost-effective [23]. The utilization of ^68^Ga-PSMA-11 PET/CT as a relatively novel imaging modality is often accompanied by higher associated costs [24]. Moreover, the limited availability of requisite infrastructure may pose challenges, particularly in regions with resource limitations [25]. While the upfront expense of implementing ^68^Ga-PSMA-11 PET/CT scans for initial assessment might be prohibitive at present [26], ongoing developments in technology and increased accessibility could mitigate these barriers over time. With ^68^Ga-PSMA-11 PET/CT becoming more commonplace, the resultant increase in demand could potentially lead to economies of scale, ultimately driving down costs in the long run [27]. Nonetheless, a comprehensive analysis is imperative to determine the potential cost-effectiveness of employing and integrating ^68^Ga-PSMA-11 PET/CT for the primary diagnosis of patients with suspected prostate cancer.

Nonetheless, the initiation of treatment for suspicion of PCa without histological confirmation remains controversial. However, it is important to note that we are not suggesting that the existing diagnostic pathway be replaced with one that entirely omits prostatic biopsy in all patients. Rather, we believe that the combined usage of both imaging modalities may provide a highly selected subset of patients with the option of omitting biopsy confirmation. Patients with fewer suspect lesions seen on the scans should still undergo further investigation with prostatic biopsy. Naturally, the difficulty will lie in selecting appropriate cut-off values to be able to risk-stratify the patients sufficiently such that we can avoid subjecting false positive cases to unnecessary prostate surgery [28]. One potential avenue for future research would be to create a predictive model that integrates both pre-biopsy parameters and the PRIMARY score [29] for the accurate prediction of csPCa. Developed from its eponymous trial, the PRIMARY score is a five-category scale devised to discern csPCa by amalgamating information pertaining to anatomical location, pattern, and intensity derived from ^68^Ga-PSMA-11 PET/CT imaging findings [29]. While existing research has predominantly focused on assessing the reproducibility and reliability of this scoring system, preliminary analyses have indicated significant promise for its utility [30]. This merits close monitoring of its future advancements and refinements.

## 5. Conclusions

^68^Ga-PSMA-11 PET/CT is accurate and complementary to mpMRI in terms of differentiating areas containing csPCa within the prostate. Our results suggest that, for a highly selected subgroup of patients with high suspicion of CsPCa indicated on mpMRI and ^68^Ga-PSMA-11 PET/CT, it may be a feasible option to skip prostate biopsy prior to undergoing RP. Further studies with prospective evaluations will be required in order to confirm these results.

## Figures and Tables

**Table 1:** Patient and Lesion Characteristics.

Variable		Overall	ISUP Grade Group ≥ 2
Number of Patients		42	42
Age, year (IQR)		69 (65.8–71.3)	"
PSA, ng/mL (IQR)		12.0 (6.08–24.3)	"
PV, mL (IQR)		46 (31.9–58.0)	"
PSA Density, ng/mL/mL (IQR)		0.253 (0.170–0.670)	"
Number of Lesions		122	92
mpMRI, *n* (%)		122	92
	Negative	3 (2.5)	2 (2.2)
	PI-RADS 3	18 (14.8)	7 (7.6)
	PI-RADS 4	65 (53.3)	48 (52.2)
	PI-RADS 5	36 (29.5)	35 (38.0)
^68^Ga-PSMA-11 PET/CT, *n* (%)		122	92
	Negative	42 (34.4)	20 (21.7)
	Positive	80 (65.6)	72 (78.3)
	SUVmax	4.45 (1.88–8.28)	5.75 (2.50–11.2)
Histology, *n* (%)		122	92
	Nil cancer	22 (18.0)	"
	3 + 3	8 (6.6)	"
	3 + 4	45 (36.9)	45 (48.9)
	4 + 3	28 (23.0)	28 (30.4)
	≥4 + 4	19 (15.6)	19 (20.7)

ISUP, International Society of Urological Pathology; *n*, the number of patients; PSA, prostate-specific antigen; PV, prostate volume; mpMRI, multiparametric magnetic resonance imaging; PI-RADS, Prostate Imaging-Reporting and Data System; ^68^Ga-PSMA-11 PET/CT, ^68^Ga-Prostate-specific membrane antigen positron emission tomography/computed tomography; SUVmax, maximum standardised uptake value.

**Table 2:** Diagnostic Accuracy of mpMRI & ^68^Ga-PSMA-11 PET/CT Independently & In Combination.

	mpMRI 4–5	^68^Ga-PSMA-11 PET/CT	mpMRI 4–5 + ^68^Ga-PSMA-11 PET/CT
Sensitivity, %	90.2 (83.1–95.2)	78.5 (69.4–86.0)	74.2 (64.7–82.4)
Specificity, %	40.0 (23.8–57.8)	73.3 (56.0–86.8)	83.3 (67.5–93.7)
PPV, %	82.2 (74.0–88.8)	90.1 (82.4–95.4)	93.2 (86.0–97.5)
NPV, %	57.1 (36.1–76.6)	52.4 (37.5–67.0)	51.0 (37.2–64.7)
AUC	0.65	0.76	0.79

mpMRI, multiparametric magnetic resonance imaging; ^68^Ga-PSMA-11 PET/CT, ^68^Ga-Prostate-specific membrane antigen positron emission tomography/computed tomography; PPV, positive predictive value; NPV, negative predictive value.

## Data Availability

The raw data supporting the conclusions of this article will be made available by the authors upon request.
